# Sex-Specific Doppler Patterns of the Umbilical Artery, Middle Cerebral Artery, and Descending Abdominal Aorta in an African Pregnancy Cohort

**DOI:** 10.7759/cureus.110982

**Published:** 2026-06-16

**Authors:** Eric Ofori, Ijeoma C Anyitey-Kokor, Andrew Donkor, Prince N Adotey, Yaw A Wiafe

**Affiliations:** 1 Medical Imaging, Kwame Nkrumah University of Science and Technology, Kumasi, GHA; 2 Radiology Directorate, Komfo Anokye Teaching Hospital, Kumasi, GHA

**Keywords:** descending abdominal aorta, doppler ultrasound, fetal sex, fetal surveillance, middle cerebral artery, umbilical artery

## Abstract

Introduction: Fetal sex is increasingly recognized as a biological variable that may influence placental development, fetal circulation, and pregnancy outcomes. However, sex-specific fetal Doppler data are limited in African populations. This study assessed whether Doppler indices of the umbilical artery (UA), middle cerebral artery (MCA), and descending abdominal aorta (DA) differ by fetal sex in a Ghanaian pregnancy cohort.

Methods: A prospective cross-sectional study was conducted among 239 low-risk singleton pregnancies attending antenatal care at the AngloGold Ashanti Health Foundation in Ghana. Fetal biometry, estimated fetal weight, fetal heart rate, and Doppler velocimetry of the UA, MCA, and DA were obtained using standardized ultrasound protocols. Fetal sex was determined antenatally and confirmed after delivery. Doppler indices were compared between male and female fetuses overall and after stratification by gestational age (≤25, 26-35, and >35 weeks) using independent-samples t-tests or Mann-Whitney U tests, with statistical significance set at p < 0.05.

Results: Male fetuses accounted for 53.6% (128/239) of the cohort, whereas female fetuses accounted for 46.4% (111/239). When all gestational ages were pooled, UA, MCA, and DA Doppler indices did not differ significantly by fetal sex. However, sex-specific patterns were observed at ≤25 weeks of gestation. Female fetuses had higher UA pulsatility index (p = 0.023), resistive index (p = 0.037), and systolic/diastolic ratio (p = 0.023), suggesting higher early fetoplacental impedance. In contrast, male fetuses had higher DA pulsatility index (p = 0.048) and resistive index (p = 0.015), suggesting higher early systemic vascular resistance. Between 26 and 35 weeks and after 35 weeks, most Doppler differences were not statistically significant. Male fetuses had higher fetal heart rates after 35 weeks (144 ± 8.67 vs 140 ± 8.92 bpm, p = 0.035).

Conclusions: In this Ghanaian cohort, fetal sex was not associated with significant overall differences in UA, MCA, or DA Doppler indices, but gestational age-specific differences were observed in early pregnancy. Female fetuses showed higher early UA impedance, whereas male fetuses showed higher early DA impedance and higher fetal heart rates in late gestation. These findings support consideration of fetal sex as a biological variable in Doppler-based fetal surveillance and highlight the need for population-specific reference data in African cohorts.

## Introduction

It is established that fetal sex is not merely a demographic label recorded during pregnancy and confirmed after birth, but a biological variable that plays a role in shaping pregnancy from the earliest stages of development. Pregnancy outcomes differ systematically between male and female fetuses. Male fetuses are more often associated with preterm birth, higher birth weights, labor dystocia, cord complications, and cesarean delivery, whereas female fetuses appear more resilient in some perinatal settings [1].

Fetal sex has been found to affect several pregnancy-related parameters within a single pregnancy, including glucose metabolism, hypertensive disorders, fetal growth, placental biology, fetal monitoring, environmental responses, and even maternal health after pregnancy [2]. Consequently, pregnancy is a dialogue between three biological actors: the mother, the placenta, and the fetus, with fetal sex influencing the tone of that dialogue.

Male fetuses have been observed to grow larger and faster, which may help explain their higher birth weights and greater rates of macrosomia [3,4]. However, this tendency comes with costs. Faster growth demands more from the placenta and mother, and it may reduce the buffer available when pregnancy is stressed by inflammation, metabolic strain, poor placental function, or labor. Baines and West [5] provide a mechanistic explanation for this phenomenon, stating that male fetuses and placentas prioritize proliferation and nutrient metabolism, whereas female fetuses appear more adaptable and more tightly regulated. On the other hand, female-bearing pregnancies have their own risk profile. Female fetal sex is linked in several studies with maternal hypertensive disorders and altered maternal insulin resistance, including a higher risk of type 2 diabetes later in life among women who had gestational diabetes and delivered a girl [6].

The recent study by Parisi et al. [7] further reports that fetal sex is already reflected in first-trimester placental and vascular markers before birth outcomes become apparent. Female fetuses showed lower uterine artery resistance and higher serum β-hCG levels, whereas male fetuses later showed greater birth weight and lower cord pH under labor conditions. This supports the idea that the roots of sex-specific pregnancy outcomes are established early, not only at delivery.

Awareness of fetal sex, therefore, is not merely a matter of curiosity but a potential pregnancy surveillance tool. To facilitate a deeper and more contextual understanding of this subject, prenatal ultrasound and fetal Doppler assessment provide a live, repeatable, vessel-specific window into fetal and placental physiology. Not only is prenatal ultrasound highly accurate in fetal sex determination [8], but it may also be useful in shaping our understanding of the vascular pathways leading to those outcomes. The umbilical artery (UA) reflects placental impedance, the middle cerebral artery (MCA) reflects cerebral adaptation, and the descending abdominal aorta (DA) reflects systemic fetal circulation. In this regard, Doppler ultrasound becomes the bridge between the biological theory of fetal sex dimorphism and the clinical reality of pregnancy surveillance. Yet, there is a paucity of data relating fetal sex to Doppler indices. This study was therefore conducted as a baseline evaluation of the relationship between fetal sex and Doppler patterns in an African population.

## Materials and methods

Study design

This study employed a prospective cross-sectional design. It was conducted at the AngloGold Ashanti Health Foundation (AGAHF), Obuasi Mine, Ghana, a healthcare facility providing antenatal services to the Obuasi population.

Study population

Pregnant women attending routine antenatal ultrasound examinations at the study site were screened for eligibility. A total of 239 participants who met the eligibility criteria and had complete Doppler data were included in the analysis. Inclusion criteria were African descent, viable singleton pregnancy, attendance for routine antenatal ultrasound, reliable gestational age assessment using the last menstrual period corroborated by first-trimester crown-rump length or early dating information, absence of a known history of adverse pregnancy outcomes at enrollment, technically acceptable Doppler waveforms from the study vessels, antenatal fetal sex assignment, and postdelivery sex confirmation. Exclusion criteria were multiple gestation, known major pregnancy complications or adverse pregnancy history such as preeclampsia, inability to obtain interpretable UA, MCA, or DA Doppler waveforms, incomplete essential study variables, or absence of postdelivery sex confirmation. Additional exclusion criteria included known maternal medical or obstetric conditions that could independently influence fetoplacental or fetal Doppler indices, including hypertensive disorders of pregnancy, preexisting or gestational diabetes, other metabolic disorders, obesity, and other clinically documented high-risk pregnancy conditions. These criteria were applied to maintain a relatively homogeneous low-risk cohort and to minimize confounding from maternal or pregnancy-related conditions known to affect fetal Doppler velocimetry.

Sample size estimation

An a priori power analysis was conducted using G*Power version 3.1.9.7 (Heinrich Heine University Düsseldorf, Düsseldorf, Germany) to estimate the minimum sample size required to compare continuous Doppler indices between male and female fetuses. The analysis was based on an independent-samples t-test, assuming a two-sided alpha level of 0.05, 80% statistical power, equal allocation between groups, and a medium effect size (Cohen's d = 0.50). Under these assumptions, the minimum required sample size was 128 fetuses, comprising 64 male and 64 female fetuses. The final analytic sample included 239 participants with complete essential measurements and postdelivery fetal sex confirmation, exceeding the minimum required sample size.

Recruitment and consent

A purposive sampling method was employed to recruit eligible participants. This approach was selected because the study required a defined low-risk singleton pregnancy subgroup with technically adequate UA, MCA, and DA Doppler measurements during routine antenatal care. Because Doppler indices may be influenced by other pregnancy-related factors, including hypertensive disease, diabetes, and fetal growth restriction, this approach was intended to minimize avoidable clinical heterogeneity and reduce the influence of major confounding conditions.

Pregnant women attending routine antenatal clinics were approached during scheduled ultrasound visits, screened against the eligibility criteria, and enrolled if they consented and the required measurements could be obtained. Participants were informed about the study before data collection. Individuals who agreed to participate signed an informed consent form before enrollment. Where necessary, language barriers were addressed using an interpreter.

Ultrasound examination and data collection

All participants underwent standard obstetric ultrasound using a Philips ClearVue 650 system with a 2-5 MHz curvilinear transducer. Fetal biometry was performed after confirming fetal viability, placental location, and amniotic fluid volume. Estimated fetal weight (EFW) was calculated using the Hadlock IV formula based on fetal biometric parameters [9]. Gestational age was determined from the last menstrual period and confirmed by first-trimester crown-rump length or available early dating information. Fetal sex was assigned antenatally only when the external genitalia were visualized with sufficient confidence during the ultrasound examination. Postdelivery sex confirmation was performed using the recorded newborn sex in delivery or postnatal records. Doppler acquisition was performed in accordance with the International Society of Ultrasound in Obstetrics and Gynecology (ISUOG) Practice Guidelines on the Use of Doppler Velocimetry in Obstetrics [10]. To minimize the inherent operator dependence of Doppler ultrasound, all examinations were performed using a standardized protocol by a duly certified sonographer with more than 10 years of experience in obstetric Doppler velocimetry. Doppler ultrasound was performed with the mother in the supine position. Waveforms were recorded over at least two cardiac cycles using a 2-mm sample volume and a medium wall filter. Measurements were obtained from the UA (free-floating loop), MCA, and DA (above the renal arteries). Only technically acceptable waveforms were included in the analysis, and examinations with unclear, uninterpretable, or technically inadequate Doppler findings were excluded in accordance with the study eligibility criteria. These measures were intended to reduce measurement variability and minimize the influence of technical acquisition difficulties on the observed Doppler patterns.

Data management and statistical analysis

Data were entered into Microsoft Excel for Microsoft 365 (Microsoft Corporation, Redmond, Washington) for cleaning and coding and then imported into jamovi version 2.7.24 (The jamovi Project; released 2026) for statistical analysis [11]. Continuous variables were summarized using mean ± standard deviation to align with the Doppler comparison tables, whereas distributional shape was evaluated before group testing. Normality was assessed using the Kolmogorov-Smirnov test. Normally distributed variables were compared between male and female fetuses using independent-samples t-tests, with mean differences and 95% confidence intervals reported where applicable. Nonnormally distributed variables were compared using the Mann-Whitney U test; p-values for these variables were interpreted together with the descriptive statistics and confidence intervals. All tests were two-tailed, and statistical significance was set at p < 0.05.

Ethical considerations

Ethical approval was obtained from the AGAHF Ethical Review Board. Informed consent was obtained from all participants. Participation was voluntary, and confidentiality was maintained using unique identifiers. Data were stored securely and were accessible only to authorized personnel.

## Results

A total of 239 participants were included in the study. Of these, 98 (41.0%) had a gestational age greater than 35 weeks, 80 (33.5%) were between 26 and 35 weeks, and 61 (25.5%) were at or below 25 weeks' gestation. Overall, 71.1% (n = 170) had fetuses in cephalic presentation, 23.8% (n = 57) had breech presentation, and 5% (n = 12) had transverse presentation. The distribution of fetal sex showed a slight predominance of males, 53.6% (n = 128), compared with females, 46.4% (n = 111). Table 1 presents the characteristics of the study participants.

**Table 1:** Characteristics of study participants (n = 239). Percentages are based on the total study population unless otherwise stated and may not sum to 100.0% because of rounding. Gestational-age groups are labeled explicitly in weeks. Percentile/reference-range data were not present in the source table; if a reference-range table is added, label percentile columns as 5th, 50th, and 95th percentile for each gestational-age stratum. EGA: estimated gestational age, EFW: estimated fetal weight, FHR: fetal heart rate, SD: standard deviation, n: frequency.

Variable	Category or stratum	n	%	Mean	SD	Minimum	Maximum
Maternal age (years)	Overall			29.1	5.73	16	44
Estimated gestational age (weeks)	Overall			30.8	6.54	15	40
EGA group (weeks)	≤25	61	25.5				
EGA group (weeks)	26-35	80	33.5				
EGA group (weeks)	>35	98	41.0				
Estimated fetal weight (g)	Overall			1988	1165	112	4877
Fetal heart rate (bpm)	Overall			144	21.4	122	439
Presentation	Breech	57	23.8				
Presentation	Cephalic	170	71.1				
Presentation	Transverse	12	5.0				
Placentation	Fundal	20	8.4				
Placentation	Posterofundal	9	3.8				
Placentation	Anterior	111	46.8				
Placentation	Posterior	87	36.7				
Placentation	Previa	8	3.4				
Placentation	Anterofundal	2	0.8				
Fetal sex	Female	111	46.4				
Fetal sex	Male	128	53.6				

Doppler parameters

**Figure 1:**
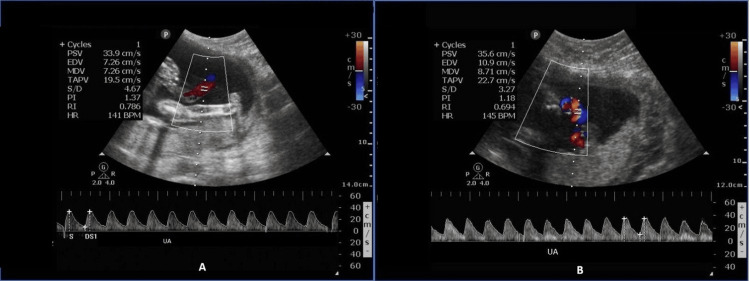
: Umbilical artery waveform for female (A) and male (B) fetuses at 21 weeks.

**Figure 2:**
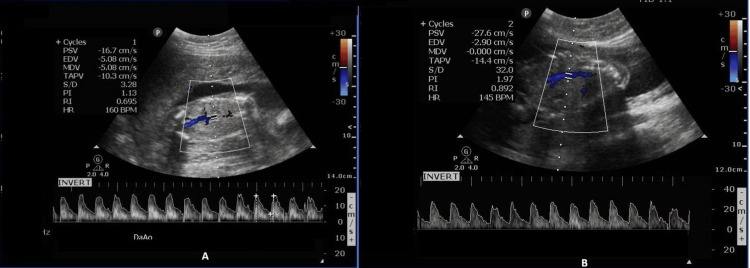
Descending abdominal aorta waveform for female (a) and male (b) fetuses at 23 weeks.

Table 2 presents the descriptive statistics for all Doppler parameters measured among the study participants. The mean UA pulsatility index (PI) and resistive index (RI) were 1.18 ± 0.32 (range, 0.56-2.08) and 0.65 ± 0.10 (range, 0.41-0.872), respectively. The mean MCA pulsatility index and resistive index were 1.12 ± 0.63 and 0.77 ± 0.08, respectively. The mean DA pulsatility index and resistive index were 1.69 ± 0.33 and 0.79 ± 0.07, respectively. All measured Doppler parameters except the UA resistive index (RI) were nonnormally distributed (p < 0.05). Figure 1 and Figure 2 are representative images of the UA and the DA, respectively, showing differences in fetal Doppler indices between females and males at less than 25 weeks' gestational age. 

**Table 2:** Descriptive statistics of Doppler parameters. p-value <0.05 signifies non-parametric parameter; Bold indicates p ≥ 0.05. SD: standard deviation; Min: minimum; Max: maximum; KS: Kolmogorov-Smirnov.

Parameters	Mean ± SD	Range (Min-Max)	KS p-value
Umbilical artery			
Peak systolic velocity	41.60 ± 10.80	17.1-71.8	0.045
​​​​​​​End diastolic velocity	14.90 ± 6.19	3.87-37.1	0.001
​​​​​​​Pulsatility index	1.18 ± 0.32	0.56-2.08	<0.001
​​​​​​​Resistive index	0.65 ± 0.10	0.41-0.872	0.063
​​​​​​​Systolic/diastolic	3.89 ± 12.00	1.68-7.83	<0.001
Middle cerebral artery			
​​​​​​​Peak systolic velocity	44.4 ± 15.80	13.6-93.2	<0.001
​​​​​​​End diastolic velocity	9.93 ± 4.78	3.23-26.6	<0.001
​​​​​​​Pulsatility index	1.12 ± 0.63	-1.08-3.44	<0.001
​​​​​​​Resistive index	0.77 ± 0.08	0.54-0.93	0.008
Descending abdominal aorta			
​​​​​​​Peak systolic velocity	41.9 ± 14.9	15.0-90.40	<0.001
​​​​​​​End diastolic velocity	8.37 ± 2.81	3.23-19.40	<0.001
​​​​​​​Pulsatility index	1.69 ± 0.33	0.85-3.00	0.023
​​​​​​​Resistive index	0.79 ± 0.07	0.55-0.90	<0.001

Comparison of parameters between fetal sex

Tables 3-5 present data comparing all measured Doppler parameters by fetal sex across estimated gestational age strata, whereas Table 6 compares fetal heart rates. At 25 weeks' gestation and below, there were significant differences in UA PI, RI, and S/D ratio between male and female fetuses, with females having higher indices than males (p < 0.05). At the same gestational age stratum, descending abdominal aorta PI and RI also differed significantly between the two sexes, with males having higher indices than females (PI: p = 0.048; RI: p = 0.015). 

**Table 3:** Umbilical artery Doppler parameters by fetal sex and gestational age group. Overall sex distribution: male, n = 128 (53.6%); female, n = 111 (46.4%). EGA n (%) indicates the total stratum size: total sample, n = 239 (100.0%); ≤25 weeks, n = 61 (25.5%); 26-35 weeks, n = 80 (33.5%); and >35 weeks, n = 98 (41.0%). All p-values and 95% CIs are transcribed from the source table. p-values < 0.05 are bolded. Statistical tests should be stated in the manuscript and table note as follows after final confirmation: normally distributed variables were compared using the independent-samples t-test or Welch t-test, as appropriate; nonnormally distributed variables were compared using the Mann-Whitney U test with the Hodges-Lehmann estimate and 95% CI. State whether p-values are unadjusted or adjusted for multiple comparisons. EGA: estimated gestational age; SD: standard deviation; MD: mean difference; CI: confidence interval; bpm: beats per minute.

Parameter	EGA stratum	EGA n (%)	Male mean ± SD	Female mean ± SD	MD	95% CI	p-value
Peak systolic velocity	Total sample	239 (100.0)	41.43 ± 10.58	41.81 ± 11.10	-0.50	-3.20 to 2.40	0.751
Peak systolic velocity	≤25 weeks	61 (25.5)	35.22 ± 10.15	32.67 ± 5.94	-2.00	-6.90 to 2.00	0.262
Peak systolic velocity	26-35 weeks	80 (33.5)	42.31 ± 9.76	44.85 ± 11.60	1.60	-3.70 to 7.30	0.612
Peak systolic velocity	>35 weeks	98 (41.0)	44.16 ± 10.28	46.56 ± 9.57	2.00	-2.10 to 6.30	0.311
End diastolic velocity	Total sample	239 (100.0)	15.16 ± 6.12	14.63 ± 6.28	-0.50	-2.30 to 1.10	0.583
End diastolic velocity	≤25 weeks	61 (25.5)	10.39 ± 4.54	8.09 ± 2.66	-1.74	-4.03 to 0.20	0.102
End diastolic velocity	26-35 weeks	80 (33.5)	15.08 ± 5.42	15.77 ± 4.97	0.80	-2.10 to 3.20	0.638
End diastolic velocity	>35 weeks	98 (41.0)	17.92 ± 5.88	18.80 ± 4.95	0.90	-0.80 to 3.20	0.288
Pulsatility index	Total sample	239 (100.0)	1.15 ± 0.30	1.21 ± 0.35	0.05	-0.04 to 0.13	0.275
Pulsatility index	≤25 weeks	61 (25.5)	1.38 ± 0.20	1.54 ± 0.30	0.17	0.03 to 0.30	0.023
Pulsatility index	26-35 weeks	80 (33.5)	1.17 ± 0.31	1.16 ± 0.25	0.02	-0.13 to 0.13	0.789
Pulsatility index	>35 weeks	98 (41.0)	1.01 ± 0.25	1.00 ± 0.24	-0.00	-0.10 to 0.22	0.962
Resistive index	Total sample	239 (100.0)	0.64 ± 0.09	0.66 ± 0.11	0.02	-0.01 to 0.05	0.116
Resistive index	≤25 weeks	61 (25.5)	0.72 ± 0.06	0.75 ± 0.08	0.04	0.00 to 0.07	0.037
Resistive index	26-35 weeks	80 (33.5)	0.64 ± 0.10	0.65 ± 0.08	0.00	-0.03 to 0.04	0.844
Resistive index	>35 weeks	98 (41.0)	0.59 ± 0.09	0.59 ± 0.09	0.00077	-0.03 to 0.04	0.965
Systolic/diastolic ratio	Total sample	239 (100.0)	2.99 ± 0.94	3.25 ± 1.23	0.14	-0.06 to 0.36	0.171
Systolic/diastolic ratio	≤25 weeks	61 (25.5)	3.63 ± 0.70	4.39 ± 1.33	0.69	0.09 to 1.30	0.023
Systolic/diastolic ratio	26-35 weeks	80 (33.5)	3.08 ± 1.06	3.02 ± 0.86	0.07	-0.27 to 0.34	0.637
Systolic/diastolic ratio	>35 weeks	98 (41.0)	2.57 ± 0.70	2.56 ± 0.64	0.000069	-0.18 to 0.22	0.991

**Table 4:** Middle cerebral artery Doppler parameters by fetal sex and gestational age group.

Parameter	EGA stratum	EGA n (%)	Male mean ± SD	Female mean ± SD	MD	95% CI	p-value
Peak systolic velocity	Total sample	239 (100.0)	45.00 ± 14.19	44.07 ± 17.03	-1.80	-6.00 to 2.40	0.387
Peak systolic velocity	≤25 weeks	61 (25.5)	30.04 ± 6.80	28.42 ± 6.27	-0.80	-4.60 to 2.40	0.608
Peak systolic velocity	26-35 weeks	80 (33.5)	43.91 ± 11.88	44.19 ± 14.32	-0.000035	-6.40 to 6.40	1.000
Peak systolic velocity	>35 weeks	98 (41.0)	53.87 ± 11.74	54.30 ± 16.01	0.20	-5.70 to 6.50	0.877
End diastolic velocity	Total sample	239 (100.0)	9.94 ± 4.56	9.92 ± 5.04	-0.16	-1.33 to 0.81	0.665
End diastolic velocity	≤25 weeks	61 (25.5)	6.18 ± 1.67	6.72 ± 2.55	0.00	-0.85 to 1.21	0.941
End diastolic velocity	26-35 weeks	80 (33.5)	9.30 ± 4.32	9.56 ± 5.14	-0.000029	-1.78 to 2.15	0.947
End diastolic velocity	>35 weeks	98 (41.0)	12.48 ± 4.28	12.29 ± 5.05	-0.60	-2.42 to 1.60	0.636
Pulsatility index	Total sample	239 (100.0)	1.10 ± 0.64	1.14 ± 0.63	0.02	-0.12 to 0.16	0.840
Pulsatility index	≤25 weeks	61 (25.5)	1.15 ± 0.69	1.02 ± 0.38	-0.17	-0.42 to 0.11	0.215
Pulsatility index	26-35 weeks	80 (33.5)	1.24 ± 0.68	1.36 ± 0.85	0.06	-0.26 to 0.36	0.750
Pulsatility index	>35 weeks	98 (41.0)	0.96 ± 0.55	1.06 ± 0.54	0.09	-0.10 to 0.32	0.330
Resistive index	Total sample	239 (100.0)	0.78 ± 0.07	0.77 ± 0.08	-0.01	-0.03 to 0.02	0.596
Resistive index	≤25 weeks	61 (25.5)	0.79 ± 0.06	0.77 ± 0.07	-0.02	-0.06 to 0.01	0.236
Resistive index	26-35 weeks	80 (33.5)	0.79 ± 0.07	0.78 ± 0.09	0.00	-0.04 to 0.04	0.944
Resistive index	>35 weeks	98 (41.0)	0.76 ± 0.07	0.76 ± 0.09	0.00	-0.04 to 0.04	0.943

**Table 5:** Descending abdominal aorta Doppler parameters by fetal sex and gestational age group. p-values < 0.05 are bolded.

Parameter	EGA stratum	EGA n (%)	Male mean ± SD	Female mean ± SD	MD	95% CI	p-value
Peak systolic velocity	Total sample	239 (100.0)	42.75 ± 15.13	40.83 ± 14.69	-1.60	-5.70 to 2.40	0.457
Peak systolic velocity	≤25 weeks	61 (25.5)	31.76 ± 7.93	32.49 ± 13.27	-0.82	-7.10 to 5.90	0.803
Peak systolic velocity	26-35 weeks	80 (33.5)	41.60 ± 15.39	41.97 ± 14.19	0.80	-6.40 to 7.30	0.821
Peak systolic velocity	>35 weeks	98 (41.0)	49.58 ± 14.31	46.05 ± 13.67	-3.90	-10.50 to 2.40	0.211
End diastolic velocity	Total sample	239 (100.0)	8.35 ± 3.02	8.39 ± 2.55	0.16	-0.55 to 0.81	0.522
End diastolic velocity	≤25 weeks	61 (25.5)	6.67 ± 2.46	7.50 ± 2.47	0.80	-0.73 to 2.42	0.305
End diastolic velocity	26-35 weeks	80 (33.5)	8.12 ± 2.44	8.57 ± 2.17	0.57	-0.64 to 1.62	0.278
End diastolic velocity	>35 weeks	98 (41.0)	9.44 ± 3.31	8.88 ± 2.79	-0.16	-1.61 to 0.81	0.632
Pulsatility index	Total sample	239 (100.0)	1.72 ± 0.32	1.65 ± 0.34	-0.06	-0.15 to 0.04	0.222
Pulsatility index	≤25 weeks	61 (25.5)	1.68 ± 0.23	1.53 ± 0.34	-0.18	-0.32 to 0.00	0.048
Pulsatility index	26-35 weeks	80 (33.5)	1.71 ± 0.35	1.65 ± 0.32	-0.02	-0.19 to 0.14	0.763
Pulsatility index	>35 weeks	98 (41.0)	1.74 ± 0.34	1.73 ± 0.34	-0.02	-0.17 to 0.15	0.808
Resistive index	Total sample	239 (100.0)	0.80 ± 0.07	0.78 ± 0.08	-0.01	-0.03 to 0.01	0.197
Resistive index	≤25 weeks	61 (25.5)	0.80 ± 0.05	0.75 ± 0.08	-0.05	-0.09 to -0.01	0.015
Resistive index	26-35 weeks	80 (33.5)	0.79 ± 0.07	0.78 ± 0.08	-0.01	-0.04 to 0.02	0.627
Resistive index	>35 weeks	98 (41.0)	0.78 ± 0.08	0.80 ± 0.08	0.00	-0.02 to 0.03	0.814

**Table 6:** Fetal heart rate by fetal sex and gestational age group. p-values < 0.05 are bolded.

Parameter	EGA stratum	EGA n (%)	Male mean ± SD	Female mean ± SD	MD	95% CI	p-value
Fetal heart rate (bpm)	Total sample	239 (100.0)	145 ± 9.24	142 ± 9.73	-3.00	-5.00 to -0.000026	0.018
Fetal heart rate (bpm)	≤25 weeks	61 (25.5)	149 ± 9.69	145 ± 9.07	-3.00	-8.00 to 2.00	0.224
Fetal heart rate (bpm)	26-35 weeks	80 (33.5)	143 ± 8.96	140 ± 10.70	-3.00	-8.00 to 2.00	0.164
Fetal heart rate (bpm)	>35 weeks	98 (41.0)	144 ± 8.67	140 ± 8.92	-4.00	-7.00 to -0.000002	0.035

In total, there was no significant difference in any of the Doppler parameters between the two sexes (p > 0.05). From gestational ages of 26 weeks to 35 weeks, there was no significant difference in Doppler parameters between the two sexes (p > 0.05), but there were interesting disparities in mean Doppler indices. For instance, females had higher MCA PSV, EDV, and PI compared to males.

For fetuses beyond 35 weeks, there was no significant difference in Doppler parameters between males and females. However, males had higher descending aortic PSV, EDV, and PI than females. Females had higher UA PSV and EDV compared to males. In all, there was a significant difference in FHR between male fetuses and female fetuses (p = 0.018). Beyond the gestational age of 35 weeks, there was a significant difference in FHR between the two sexes, with males (144 ± 8.67) having higher heart rates than females (140 ± 8.92) (see Table 3).

Reference ranges of Doppler parameters

The reference ranges of UA RI and PI, MCA RI and PI, and DA RI and PI across the established gestational age groups are presented in Table 7. 

**Table 7:** Reference ranges of Doppler parameters across gestational age groups. GA: gestational age; UA: umbilical artery; MCA: middle cerebral artery; DA: descending abdominal aorta; CPA: cerebroplacental ratio; PI: pulsatility index; RI: resistive index.

	GA groups	UA PI	UA RI	MCA PI	MCA RI	DA PI	DA RI	CPR
2.5th percentile	Total	0.673	0.463	0.339	0.623	1.02	0.611	0.284
	≤ 25	0.992	0.597	0.398	0.658	1.08	0.630	0.308
	26 - 35	0.750	0.500	0.336	0.633	1.03	0.613	0.315
	>35	0.632	0.454	0.338	0.619	1.02	0.608	0.286
5th percentile	Total	0.713	0.486	0.385	0.637	1.12	0.644	0.341
	≤ 25	1.060	0.620	0.493	0.667	1.11	0.639	0.328
	26 - 35	0.798	0.507	0.405	0.649	1.18	0.669	0.365
	>35	0.666	0.462	0.369	0.624	1.15	0.656	0.374
10th percentile	Total	0.786	0.512	0.471	0.676	1.25	0.684	0.449
	≤ 25	1.130	0.647	0.555	0.678	1.19	0.664	0.402
	26 - 35	0.884	0.549	0.560	0.684	1.30	0.701	0.504
	>35	0.709	0.485	0.429	0.656	1.26	0.684	0.458
15th percentile	Total	0.860	0.539	0.589	0.687	1.35	0.714	0.493
	≤ 25	1.170	0.656	0.637	0.707	1.33	0.704	0.446
	26 - 35	0.909	0.563	0.643	0.696	1.38	0.725	0.547
	>35	0.750	0.500	0.473	0.679	1.37	0.720	0.509
25th percentile	Total	0.935	0.574	0.679	0.717	1.47	0.744	0.599
	≤ 25	1.250	0.684	0.782	0.727	1.45	0.740	0.533
	26 - 35	0.951	0.580	0.781	0.734	1.46	0.740	0.656
	>35	0.823	0.531	0.632	0.702	1.50	0.754	0.606
50th percentile	Total	1.140	0.646	0.985	0.779	1.69	0.796	0.844
	≤ 25	1.470	0.747	1.050	0.795	1.58	0.773	0.741
	26 - 35	1.110	0.635	1.050	0.787	1.69	0.796	1.010
	>35	1.000	0.600	0.854	0.761	1.77	0.821	0.947
75th percentile	Total	1.360	0.714	1.370	0.833	1.92	0.846	1.280
	≤ 25	1.650	0.786	1.320	0.823	1.81	0.821	0.887
	26 - 35	1.310	0.698	1.710	0.853	1.90	0.839	1.500
	>35	1.160	0.655	1.290	0.821	2.00	0.857	1.280
90th percentile	Total	1.650	0.786	1.890	0.867	2.12	0.879	1.800
	≤ 25	1.800	0.818	1.690	0.845	2.05	0.870	1.360
	26 - 35	1.560	0.766	2.330	0.874	2.10	0.871	2.110
	>35	1.280	0.691	1.760	0.863	2.15	0.885	1.770
95th percentile	Total	1.780	0.814	2.310	0.881	2.19	0.889	2.180
	≤ 25	1.880	0.833	1.840	0.854	2.15	0.884	1.540
	26 - 35	1.670	0.787	2.790	0.880	2.18	0.881	2.340
	>35	1.350	0.720	1.960	0.896	2.20	0.892	2.230
97.5th percentile	Total	1.880	0.833	2.840	0.904	2.210	0.893	2.610
	≤ 25	1.940	0.846	2.040	0.857	2.180	0.888	1.770
	26 - 35	1.910	0.839	3.230	0.902	2.210	0.890	2.850
	>35	1.680	0.798	2.240	0.907	2.210	0.894	2.600

## Discussion

This study adds an important vascular layer to the fetal sex story. The most important finding is that the overall male-female differences were not significant when all gestational ages were pooled, but sex differences emerged when the data were examined by gestational age group.

At ≤25 weeks' gestation, female fetuses had significantly higher UA PI, RI, and S/D ratio than males. Since UA Doppler reflects downstream placental vascular resistance, this suggests that, in this African cohort, female-bearing pregnancies may show relatively higher fetoplacental impedance earlier in gestation.

This is interesting because it complicates the common view that male fetuses are always the more placentally vulnerable group. It may instead indicate that male and female fetuses place different demands on the maternal-placental-fetal system at different times.

Also, at ≤25 weeks' gestation, males had significantly higher DA PI and RI than females. The DA reflects fetal systemic circulation rather than placental resistance alone. This suggests that, while females showed a stronger umbilical-placental resistance signal, males showed a stronger fetal systemic vascular resistance signal. This fits the broader hypothesis proposed by Baines and West [5]. Their review describes male fetuses and placentas as prioritizing growth, whereas female fetuses and placentas tend to show greater biosensing and adaptive regulation.

The MCA findings did not differ significantly by sex. Between 26 and 35 weeks' gestation, females had higher mean MCA PSV, EDV, and PI than males, but these differences were not statistically significant. This is important because the MCA is often interpreted as a marker of fetal cerebral redistribution or "brain-sparing." The absence of a strong MCA sex difference suggests that this population was not showing a broad sex-specific pattern of cerebral compromise. The sex differences appear more subtle: placental-side resistance in females early in gestation and systemic flow differences in males, rather than a clear pattern of cerebral redistribution. This study also showed that males had higher descending aortic PSV, EDV, and PI, although these Doppler differences were not statistically significant.

Beyond 35 weeks' gestation, males had significantly higher fetal heart rates than females (144 ± 8.67 vs 140 ± 8.92 bpm). This late male pattern is relevant to Parisi et al. [7], who found that male fetuses had higher birth weight and lower arterial cord pH after planned vaginal delivery, suggesting reduced resilience to labor stress. While cord pH or labor outcomes were not measured in this African cohort, the higher male fetal heart rate and tendency toward stronger aortic flow indices may represent a prelabor hemodynamic echo of the same broader phenomenon, namely that male fetuses may carry higher circulatory demand late in pregnancy [12].

This study is in agreement with Widnes et al. [13], who found that female fetuses had significantly higher UA PI and RI, largely during the first half of pregnancy, than male fetuses. However, Widnes et al. indicated that the female UA vascular dominance extended slightly beyond the first half of pregnancy. It is also important to note that this study agrees with both Widnes et al. [13] and Prior et al. [14] in finding no significant sex difference in UA pulsatility indices in term pregnancies. This agreement is important because the UA is a proxy for placental vascular impedance. Higher UA PI and RI generally indicate greater downstream placental resistance. Therefore, these studies suggest that, in otherwise healthy general pregnancy populations, female fetuses may have slightly higher placental-side vascular resistance than male fetuses during the earlier stages of gestation.

Additionally, this study agrees with Jagota et al. [15], who found no significant sex difference in MCA PI across gestation. The study by Prior et al. [14] documented higher MCA PI values in term female fetuses, but this difference was not statistically significant. This study also agrees with Jagota et al. [15], who reported that the trajectory of DA PI differed by fetal sex.

In general, this African study contributes by showing that similar sex-related Doppler differences can be detected in an African population, but with a pattern that is partly concordant with and partly distinct from those reported in European and North American cohorts.

Clinically, the agreement of these findings with previous Doppler studies supports their relevance as preliminary evidence for considering fetal sex in the future development of Doppler reference data, rather than as an immediate basis for clinical decision-making. In settings where fetal surveillance relies heavily on UA, MCA, DA, and cerebroplacental indices, sex-specific reference data may help reduce overinterpretation or underinterpretation of borderline Doppler findings, especially in early gestation and in populations underrepresented in existing reference datasets. The present results are therefore most useful for hypothesis generation and local reference-range development. A longitudinal study in an African population that can determine whether sex-specific Doppler differences predict growth restriction, labor intolerance, or other perinatal outcomes is also recommended [13-15]. This is important because a relatively low-risk singleton pregnancy population may differ from an unselected obstetric population, an issue that future studies should address.

These Doppler studies show that fetal sex affects fetal circulation in subtle but meaningful ways. Widnes et al. [13] highlighted higher female UA impedance, Jagota et al. [15] demonstrated sex-specific UA and DA trajectories, and Prior et al. [14] reported lower male MCA PI and umbilical venous flow at term. This African study contributes by showing that sex-specific Doppler differences are also detectable in an African population, especially during early gestational windows.

These studies provide evidence supporting the broader biological concept described by Baines and West [5] that male and female fetuses differ in growth strategy, immune signaling, placental behavior, and adaptive reserve. Doppler ultrasound provides a clinical window into this biology by showing how these differences may manifest as vascular impedance, cerebral perfusion, systemic circulation, and fetal heart rate differences.

While earlier studies have argued that fetal sex shapes placental development, maternal physiology, immune tone, fetal growth, and perinatal vulnerability, this African population suggests that sexual dimorphism can also be observed in fetoplacental and fetal systemic blood-flow patterns. This points in the same broad direction as previous fetal Doppler ultrasound studies, although the patterns are not uniform across all vessels, gestational ages, or populations.

Previous Doppler studies have shown that sex differences may appear in the UA, MCA, DA, ductus venosus, or umbilical venous flow, depending on when and how the fetus is evaluated. This African study contributes by showing that similar sex-related Doppler differences can be detected in an African population, but with a pattern that is partly concordant with and partly distinct from those reported in European and North American cohorts.

A major limitation of this study is its cross-sectional design. Because each fetus was assessed at a single time point, the study cannot establish causality, determine individual Doppler trajectories, or distinguish true developmental change from differences in the composition of gestational-age strata. Earlier studies by Widnes et al. and Jagota et al. used longitudinal designs, which are better suited for detecting sex-specific slopes or rates of Doppler change over gestation [13,15]. Because this was an observational study involving pregnant women, additional Doppler acquisitions solely for experimental reproducibility testing were not undertaken in order to avoid unnecessary fetal ultrasound exposure and to remain consistent with the ALARA principle. However, Doppler measurements were standardized according to ISUOG guidance, performed by an experienced certified sonographer, and restricted to technically acceptable waveforms to minimize operator-dependent variability. The purposive sampling strategy and final sample size may also limit generalizability. Although purposive sampling allowed recruitment of a carefully defined low-risk cohort and helped reduce heterogeneity from major maternal and pregnancy-related confounders, it may also limit applicability to unselected obstetric populations, high-risk pregnancies, or settings with different maternal and fetal characteristics.

The analysis was based primarily on unadjusted comparisons, which makes it vulnerable to type I error and should therefore be considered exploratory. However, statistically significant subgroup findings were concentrated in the ≤25-week gestational-age stratum and were not randomly distributed across all vessels or gestational-age groups. As in previous studies, female fetuses showed consistently higher UA impedance indices, including PI, RI, and S/D ratio. Conversely, male fetuses showed higher DA PI and RI. The MCA did not show significant sex-specific differences. This pattern suggests that the observed differences were vessel-specific and physiologically coherent.

## Conclusions

In conclusion, this study found sex-associated differences in selected Doppler indices, with female fetuses showing higher early UA impedance and male fetuses showing higher early descending abdominal aortic impedance. These findings add population diversity, vascular breadth, and local reference data to the growing body of evidence that fetal sex should be considered a biological variable in Doppler ultrasound research. Further longitudinal studies in African populations with outcome linkage are needed before sex-specific Doppler interpretation can be incorporated into individualized fetal surveillance.
